# From Conventional Septoplasty to Patient-Specific Cartilage Reshaping: A Systematic Review of Laser-Mediated and Electromechanical Approaches

**DOI:** 10.3390/jpm16060304

**Published:** 2026-06-03

**Authors:** Alessia Pennacchi, Elisa Raggini, Roberto Spasiano, Filippo Barucca, Matteo Trimarchi

**Affiliations:** 1 Department of Otorhinolaryngology Head & Neck Surgery, Cantonal Hospital Lugano, 6900 Lugano, Switzerland; alessia.pennacchi@eoc.ch (A.P.); elisa.raggini@eoc.ch (E.R.); roberto.spasiano@eoc.ch (R.S.); filippo.barucca@eoc.ch (F.B.); 2 Faculty of Biomedical Sciences, Università della Svizzera Italiana, 6900 Lugano, Switzerland

**Keywords:** nasal septal deviation, septoplasty, laser-mediated cartilage reshaping, electromechanical reshaping, personalized medicine, precision surgery, computational fluid dynamics, functional nasal surgery, cartilage biomechanics

## Abstract

**Background**: Energy-based techniques for septal cartilage reshaping, including laser-mediated cartilage reshaping (LCR) and electromechanical reshaping (EMR), have been investigated for more than three decades as minimally invasive alternatives to conventional septoplasty. Despite encouraging preclinical findings, their clinical translation remains limited. Their relevance to personalized medicine lies in the possibility of tailoring septal correction to individual anatomy, cartilage-dominant deformity patterns, and patient-specific functional needs. **Objective**: To systematically review the experimental and clinical evidence on LCR and EMR for nasal septal cartilage reshaping and to evaluate their potential role within a personalized, function-oriented treatment framework. **Methods**: A PRISMA 2020-compliant systematic search of PubMed, Scopus, and Web of Science was performed for English-language studies published up to June 2025. Original experimental or clinical studies investigating LCR or EMR applied to human or animal septal cartilage were included. Because of heterogeneity in models, dosimetry, and outcome reporting, a qualitative synthesis was undertaken. A design-specific critical appraisal was also performed. **Results**: Eighteen studies met the inclusion criteria (LCR: *n* = 9; EMR: *n* = 9). LCR consistently induced shape change within a narrow thermo-mechanical relaxation range (55–75 °C), but showed dose-dependent risks of chondrocyte injury and matrix degeneration, with limited applicability to complex osseocartilaginous deviations. Only three human LCR studies were identified, all short-term and limited to mild cartilaginous deformities. EMR produced reproducible, charge-dependent reshaping at near-physiological temperatures, but human studies were lacking. Neither technique addressed bony deviations, and none assessed functional benefit using computational fluid dynamics. **Conclusions**: Both techniques remain predominantly preclinical and may currently serve only as adjunctive options for selected anterior, cartilage-dominant deformities. Future translation will require validated dosimetry, long-term human data, and patient-specific functional assessment.

## 1. Introduction

Nasal septal deviation (NSD) is one of the most common causes of nasal obstruction and remains a leading indication for otolaryngologic evaluation. Structural alterations of the septum can impair nasal airflow and contribute to sleep disturbance, exercise intolerance, and reduced quality of life. NSD has been classified using several systems, with a reported prevalence of up to 86.6% in the general population, although symptom severity varies widely. For patients unresponsive to medical therapy, surgical correction through septoplasty remains the standard of care [1].

Septoplasty has evolved substantially over the past century. Early techniques relied on extensive cartilage resection at the expense of structural support, while later approaches—including the Cottle maneuver and contemporary structural septoplasty—aimed to correct deformities while preserving dorsal and caudal integrity. The introduction of endoscopic septoplasty improved visualization and precision, especially for posterior deviations, and resulted in lower complication rates, shorter operative times, and superior patient-reported outcomes such as NOSE score improvements [2,3,4].

Despite these advancements, conventional septoplasty still faces limitations. Cartilage manipulation is inherently mechanical and sometimes unpredictable; deformities may recur, and outcomes depend heavily on surgeon experience and the biomechanical properties of the septum. These limitations have prompted increasing interest in energy-based cartilage reshaping technologies, which aim to modify cartilage architecture while minimizing mucosal trauma and preserving tissue continuity. Among these, laser-mediated cartilage reshaping (LCR) and electromechanical reshaping (EMR) have been the most extensively studied.

In this context, the abovementioned energy-based cartilage reshaping technologies have been proposed as a paradigm shift, with the aim to induce controlled structural relaxation within cartilage, allowing shape modification without excision, scoring, or extensive reconstruction. These approaches have generated significant experimental interest over the past three decades. However, despite promising laboratory data, their translation into clinical practice has been limited. The reasons for this gap remain incompletely understood but likely involve challenges in dosimetry, biological response, and anatomical applicability, and lack of functional validation.

The aim of this systematic review is not only to summarize the available evidence on LCR and EMR, but also to critically examine why, after more than 30 years of research, these technologies have not yet translated into a viable alternative to conventional septoplasty. Modern septoplasty is still largely technique-driven, whereas energy-based reshaping could enable a shift toward patient-specific correction of selected septal phenotypes, especially when integrated with individual anatomical assessment, airflow modeling, and function-oriented planning.

## 2. Materials and Methods

### 2.1. Search Strategy

**Figure 1:**
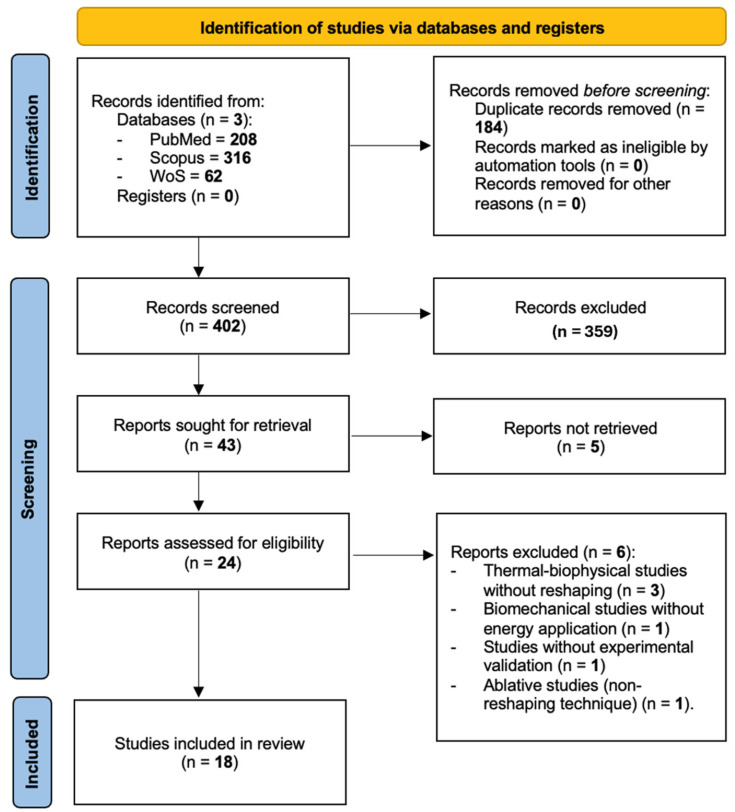
PRISMA 2020 flow diagram for study selection.

This review was conducted in accordance with PRISMA 2020 guidelines [5]; a completed PRISMA 2020 checklist is provided as Supplementary File S2, and the PRISMA 2020 flow diagram is presented in Figure 1. The full database-specific search strategies are provided in Supplementary File S1. The review protocol was not prospectively registered. A systematic search of PubMed, Scopus, and Web of Science was performed up to 30 June 2025, restricted to English-language publications from 2000 onward. The restriction to studies published from 2000 onward was applied to focus the formal synthesis on the more contemporary literature, which is more directly relevant to current translational questions, device development, and methodological reporting standards. Earlier foundational work was considered important for historical context and is acknowledged narratively, but was not included in the formal systematic synthesis. The search strategy combined the following terms: (cartilage AND (reshaping OR remodeling OR molding OR contouring)) AND (laser OR electromechanical OR thermal OR radiofrequency OR “energy-based”). Reference lists of included studies and relevant reviews were hand-searched for additional records. A design-specific critical appraisal was conducted for all 18 included studies. The three human single-arm clinical studies were appraised using the JBI Critical Appraisal Checklist for Case Series, the only in vivo animal study was assessed using SYRCLE risk-of-bias domains, and the 14 ex vivo experimental studies were evaluated using a prespecified domain-based framework tailored to bench cartilage-reshaping research. For the ex vivo studies, the following domains were assessed: adequacy of tissue source and specimen handling, standardization of specimen size/thickness and deformation model, appropriateness of control conditions, clarity and reproducibility of energy-delivery or electrode parameters, objectivity and reproducibility of outcome assessment, completeness of sample-size/replicate reporting, and appropriateness of statistical analysis. Because no validated critical appraisal tool currently exists for ex vivo septal cartilage reshaping experiments, the domain-based framework used in this review was developed de novo by the authors to capture the methodological features most relevant to reproducibility and internal validity in bench studies. The selected domains reflected recurring design issues identified across the included literature, including specimen handling, sample standardization, control conditions, protocol reproducibility, outcome objectivity, completeness of replicate reporting, and statistical transparency. Overall concern levels were assigned qualitatively: studies were judged as having moderate concerns when most domains were adequately addressed with only limited unclear reporting, whereas studies were categorized as having moderate-to-high concerns when multiple key domains were insufficiently reported or unclear, particularly in relation to controls, replicate accounting, blinding, and statistical methods. A conservative approach was applied throughout: when methodological details were not explicitly reported, items were rated as unclear rather than assumed to be adequate.

### 2.2. Study Selection

Study selection was conducted independently by two reviewers (ER and MT) using a three-step process: (1) title screening, (2) abstract screening, and (3) full-text screening. Both reviewers independently assessed all records retrieved from the database searches at each stage. Discrepancies between reviewers were resolved through discussion until consensus was reached.

### 2.3. Eligibility Criteria

Studies were selected following a PICOS framework and included if they met the following criteria:

- Population: Human or animal septal cartilage (ex vivo or in vivo).
- Intervention:
  - LCR: Non-ablative laser energy (e.g., Nd:YAG, diode, Er:Glass) combined with mechanical deformation;
  - EMR: Low-voltage direct current delivered via needle or surface electrodes combined with mechanical deformation.
- Outcomes: Objective cartilage reshaping (bend angle, shape retention, mechanical changes) and/or biological responses (viability, thermal/pH thresholds).
- Study Type: Original research with extractable data.

No formal comparator was required for inclusion, as most eligible studies were preclinical and non-comparative; conventional septoplasty was considered the clinical reference standard when interpreting translational relevance.

The exclusion criteria were as follows:

- Absence of mechanical deformation (e.g., uniform heating alone).
- Ablative techniques (e.g., coblation, laser vaporization).
- Studies limited to biomechanics or thermal characterization without reshaping.
- Numerical or finite-element simulations without experimental validation.
- Reviews, conference abstracts, editorials, or studies lacking extractable data.

Studies of scientific relevance but without reshaping were excluded from the primary analysis and considered qualitatively in the broader mechanistic interpretation.

### 2.4. Data Collection and Synthesis

For each included study, we collected data on publication year, species, model type, sample size, intervention parameters (laser dosimetry or EMR voltage/current/electrode geometry), and outcomes. Due to heterogeneity in models, dosimetry, and outcome reporting, a qualitative synthesis was performed without meta-analysis. Studies were grouped into LCR and EMR cohorts. Where sample size or follow-up/assessment timeframe were not explicitly reported in the source study, these entries were recorded as not reported (NR).

## 3. Results

### 3.1. Study Selection

The database search yielded 586 records. After removal of 184 duplicates, 402 titles/abstracts were screened. Twenty-four full-text articles were assessed, of which 18 met the inclusion criteria. These were categorized into: laser-mediated cartilage reshaping (LCR) (*n* = 9) [6,7,8,9,10,11,12,13,14] and electromechanical reshaping (EMR) (*n* = 9) [15,16,17,18,19,20,21,22,23] (PRISMA 2020 flow diagram in Figure 1).

Overall, the methodological robustness of the included evidence was limited and the body of literature was dominated by preclinical, non-comparative studies using surrogate laboratory outcomes. The three human studies fulfilled several appraisal domains, particularly with regard to procedural description and pre-/post-clinical outcome assessment, but all remained at moderate or high risk of bias because they were uncontrolled case series with no blinding and limited information on consecutive recruitment; reporting was especially sparse in the conference-format study by Sobol et al. [13]. The single in vivo animal study by Karam et al. [9] was strengthened by paired controls, long-term retrieval, objective histologic, viability, and mechanical endpoints, and ethical oversight, but most SYRCLE domains related to sequence generation, allocation concealment, random housing, blinding, and selective outcome reporting remained unclear, resulting in an overall unclear-to-high risk of bias. Among the 14 ex vivo studies, internal validity was generally moderate for mechanistic or proof-of-concept purposes but limited for direct clinical inference: common strengths included standardized specimen preparation, use of matched or concurrent controls in many studies, and objective measurements such as bend angle, temperature, viability imaging, proteoglycan assays, pH mapping, electrical parameters, histology, and mechanical testing; recurrent weaknesses included incomplete sample-size or replicate reporting, absence of blinded outcome assessment, sparse accounting for missing specimens, and abbreviated methods in several conference papers. The most recent Jahankir study showed comparatively stronger ex vivo reporting, with replicated dosimetry groups and multimodal structural, histologic, and mechanical assessment, but it remained limited by its ex vivo design and lack of blinding [23].

Taken together, the overall body of evidence was judged to be methodologically heterogeneous and at predominantly unclear or moderate-to-high risk of bias, with the main limitations being incomplete reporting, design-related non-comparativeness, and reliance on surrogate experimental endpoints rather than long-term functional clinical outcomes. Detailed critical appraisal results are shown in Table 1 and Supplementary Table S1.

### 3.2. Laser-Mediated Cartilage Reshaping (LCR)

Nine studies investigated LCR using rabbit (*n* = 2) [9,11], porcine (*n* = 4) [6,7,8,10], and human septal cartilage (*n* = 3) [12,13,14]. Sample sizes ranged from small ex vivo models (*n* = 3) [8,9,10] to a large prospective clinical study enrolling 170 patients [13]. LCR protocols employed non-ablative infrared laser sources—most frequently Nd:YAG 1.32 μm [6,7,8,9,10,11] or Er:Glass 1.56 μm [12,13,14]—delivered during mechanically imposed deformation.

Across preclinical models, effective reshaping occurred when cartilage reached 55–75 °C, corresponding to the thermo-mechanical stress relaxation zone. Within this range, studies demonstrated measurable bend-angle reduction, partial preservation of chondrocyte viability, and short- to mid-term shape retention. In contrast, higher temperatures or prolonged exposures resulted in proteoglycan loss, calcification, and structural weakening, underscoring a narrow therapeutic window [8,9,10,11].

Human studies using Er:Glass lasers reported improvements in nasal obstruction (NOSE scores) and objective airflow resistance without major complications. Nevertheless, long-term structural outcomes, viability assessment, and correction of complex deviations were not evaluated [12,13,14].

Overall, LCR can induce controlled thermo-mechanical relaxation, but reshaping outcomes depend critically on temperature control, dosimetry, and tissue characteristics (Table 2).

### 3.3. Electromechanical Reshaping (EMR)

Seven studies involved rabbit cartilage [16,17,18,19,20,21,22], one porcine model [15] and one goat model [23]. Sample size reporting was inconsistent [18,21]. EMR protocols used low-voltage direct current (1–10 V) applied during mechanical deformation through needle, plate, or strip electrodes.

Reshaping was systematically voltage- and charge-dependent, with higher charge transfer increasing bend-angle correction. Importantly, EMR operated at near-physiological temperatures, producing minimal thermal rise (<3–5 °C), consistent with a non-thermal electrochemical mechanism involving localized pH shifts and redox reactions. This was evidenced by pH-sensitive colorimetric responses and distinct electrochemical transition zones [20,22].

Viability loss was localized, typically greater near the anode at higher charges. Needle electrodes enabled deeper current penetration but introduced mechanical trauma, while newer strip-electrode designs demonstrated improved shape retention and a more favorable viability profile [19,23].

Overall, EMR provides reproducible, dose-dependent reshaping with minimal thermal injury but shows variability linked to electrode geometry, current distribution, and tissue conditions (Table 3).

## 4. Discussion

This systematic review synthesized the current evidence on two non-ablative modalities designed to reshape septal cartilage—LCR and EMR—with the aim of determining their feasibility, safety, and potential role in functional septoplasty. Although both techniques emerged in the 1990s and have been extensively evaluated in ex vivo and animal models, the translation toward human clinical practice has progressed slowly. The findings across the included studies highlight a common theme: while cartilage reshaping is consistently achievable, challenges in dosimetry, biological response, anatomical relevance, and device design currently prevent their widespread surgical adoption.

LCR and EMR share the conceptual objective of inducing targeted relaxation within the cartilage matrix to maintain a new shape under externally applied deformation: LCR achieves this via temperature-driven stress relaxation, while EMR produces a similar effect through electrochemical modification at near-physiological temperatures.

Despite these mechanistic differences, both technologies ultimately aim to offer a minimally invasive correction for selected anterior deviations, potentially widening the therapeutic portfolio of rhinologists by reducing the trauma associated with traditional cartilage excision or scoring. However, the applicability of both modalities is constrained by fundamental anatomical and technical limitations. A central issue emerging from the literature is that most studies focus on geometric reshaping rather than functional outcome. Bend angles, histologic changes, and viability assays are consistently reported, yet none of the included studies evaluated whether reshaping translated into meaningful improvement in nasal airflow or patient symptoms beyond limited short-term observations. This disconnect between structural modification and functional benefit represents a major limitation of the current evidence base.

### 4.1. Laser-Mediated Cartilage Reshaping (LCR)

Across animal and human studies, LCR consistently generated shape change when tissue temperature reached the 55–75 °C thermo-mechanical relaxation range. Within this zone, collagen fibers soften and internal stresses dissipate, allowing cartilage to adopt a new configuration. Although this principle is robust in controlled laboratory settings, several factors limit its clinical translation.

First, the thermal window is narrow and unforgiving. Minor deviations in laser power, spot size, exposure time, or tissue hydration may push temperatures beyond the therapeutic threshold, leading to chondrocyte apoptosis, matrix degeneration, and calcification. Preclinical studies showed dose-dependent decreases in viability and delayed structural compromise at higher fluences, raising concerns about long-term tissue stability.

Second, current LCR systems lack integrated real-time feedback. Only a few studies incorporated temperature sensors or control algorithms, and none demonstrated a fully closed-loop system suitable for endonasal manipulation. This absence of reliable dosimetry compromises reproducibility and limits safe clinical implementation [6,7].

Third, human septal deviation often involves the bony septum or the osseocartilaginous junction, regions that are unlikely to be adequately corrected by LCR alone and would generally still require conventional structural techniques. The dorsal L-strut, a key functional component, is also beyond the reach of predictable thermo-mechanical modification. Human trials of LCR, although encouraging in symptom improvement and safety, were limited to mild cartilaginous deviations and did not address more complex clinical scenarios.

Another factor slowing translation is the uncertain biological response following laser exposure. Although short-term studies showed partial chondrocyte survival, histological data revealed variable patterns of thermal necrosis, proteoglycan loss, and delayed structural weakening, suggesting potential long-term instability [8,9,10,11]. Human studies, while encouraging in terms of symptom improvement, lacked biological endpoints such as viability assays, long-term cartilage integrity, or imaging-based structural follow-up [12,13,14].

Finally, logistical barriers persist. High-power infrared lasers are costly, require specialized safety measures, and are not readily adaptable to confined endonasal spaces without miniaturized fiber optics, reliable heat shielding, and ergonomic instrumentation.

Although the available studies are too heterogeneous to support formal pooled dosimetric recommendations, they do allow a preliminary research-oriented synthesis. Across the LCR literature, effective reshaping appears to depend less on a single device setting than on achieving a controlled thermo-mechanical relaxation window, generally around 55–75 °C, while avoiding excessive thermal spread. From a translational perspective, future studies should therefore prioritize closed-loop temperature control, standardized reporting of surface and intrachondral temperature, exposure duration, spot geometry, tissue thickness, and post-treatment viability. Rather than pursuing higher fluence to maximize immediate bend correction, a more rational strategy would be to aim for the lowest energy delivery capable of reproducibly reaching the relaxation threshold while preserving matrix integrity. In this sense, the current literature supports a temperature-targeted dosimetric framework, rather than a device-specific one, as the most appropriate basis for future LCR optimization.

Taken together, these challenges explain why LCR, despite promising results, is unlikely to replace structural septoplasty, but may eventually find a role in carefully selected indications or as an adjunct within hybrid correction strategies.

### 4.2. Electromechanical Reshaping (EMR)

EMR modifies cartilage via low-voltage direct current that produces localized electrochemical reactions, redox gradients, and pH shifts while avoiding significant heat generation. This non-thermal mechanism provides several theoretical advantages, including decreased risk of thermal necrosis and the possibility of reshaping cartilage in mucosal environments where temperature monitoring is more difficult.

Across preclinical studies, EMR demonstrated a clear charge-dependent response: induced shape change increased with higher voltage or longer application time. The tissue warmed only minimally, reinforcing the electrochemical nature of the process. However, this same mechanism introduces complications of its own.

The first major limitation is electrode-induced injury. Chondrocyte death typically clusters around the anode and correlates with charge density, electrode penetration, and tissue conductivity. Needle electrodes can cause focal mechanical trauma, whereas plate or strip electrodes generate more uniform but superficially confined current fields. The lack of standardized electrode configurations makes optimization difficult and reduces comparability across studies [23].

Second, EMR has not yet been evaluated in human subjects. Only one animal study assessed multi-day shape retention in vivo, and long-term biological responses—such as fibrosis, inflammatory remodeling, or structural weakening—remain poorly understood [20,22,23].

Third, like LCR, EMR primarily targets cartilage rather than bone, which limits its stand-alone applicability in mixed or osseocartilaginous deformities and makes it more suitable for mild to moderate cartilage-dominant deviations. The absence of clinical-grade intranasal EMR devices also represents a major translational barrier.

A similarly cautious synthesis can be proposed for EMR. Although study designs, cartilage models, and electrode geometries vary considerably, the available evidence suggests that useful reshaping is generally achieved within a moderate charge-delivery range, whereas tissue injury increases disproportionately at higher voltages and longer application times. The rabbit ex vivo literature repeatedly indicates that clinically relevant bend correction begins to emerge around 4–6 V, while higher settings are more consistently associated with focal injury, especially near the anode. In addition, the comparative data suggest that future device development should move away from highly traumatic multi-needle configurations whenever possible and toward electrode geometries that provide more uniform current distribution with less mechanical injury, such as refined strip or surface-based designs. These observations should not be interpreted as clinical treatment recommendations, but they do provide a preliminary engineering framework for future EMR studies: moderate-voltage, charge-limited protocols combined with less traumatic and more spatially controlled electrode architectures.

On the positive side, EMR is substantially less expensive and technically simpler than LCR, requiring only a low-voltage power source. For this reason, if optimized, EMR may eventually be more amenable to office-based adoption for selected contour corrections.

### 4.3. Shared Limitations and the Persistence of Conventional Septoplasty

Both LCR and EMR, despite two fundamentally different energy delivery methods, face similar obstacles:

- Dominance of animal models: after three decades of investigation, only three human LCR studies exist, and none for EMR. The translational gap remains wide.
- Restricted applicability in mixed deformities: real-world NSD often involves complex three-dimensional, posterior, or osseocartilaginous deviations requiring mechanical repositioning or bony correction, which would generally still necessitate conventional structural techniques even if reshaping were used as an adjunct.
- Lack of long-term structural and functional outcomes: shape retention, cartilage integrity, mucosal recovery, and airflow improvement have not been systematically evaluated beyond short-term timelines.
- Absence of clinically engineered devices: neither LCR nor EMR currently offers a ready-to-use system designed specifically for endoscopic septal surgery.

These factors collectively explain the continued dominance of conventional septoplasty, which provides predictable correction of both cartilage and bone with proven long-term outcomes [24].

The predominantly moderate-to-high concerns identified in the appraisal further limit confidence in the translational readiness of these approaches.

Ethical and logistical barriers also help explain why the limited human literature is methodologically weak. High-quality randomized controlled trials in septal reshaping are difficult to design because early-phase studies must balance innovation against the risk of undertreating symptomatic nasal obstruction or exposing patients to poorly validated dosimetry. In addition, the anatomic heterogeneity of septal deviation complicates standardization of patient selection, while blinding is inherently difficult in device-based interventions that differ procedurally from conventional septoplasty. From a logistical standpoint, the small candidate population for isolated cartilage-dominant deviations, the absence of clinically engineered intranasal devices, the need for specialized training and equipment, and the challenge of combining structural, symptomatic, and functional endpoints all limit the feasibility of robust comparative trials. These factors do not justify the methodological limitations of the published clinical studies, but they do help explain why the available human evidence remains sparse, uncontrolled, and short-term.

An additional methodological limitation is that the review protocol was not prospectively registered. Although the review was conducted according to PRISMA 2020 and followed predefined eligibility and appraisal criteria, the absence of protocol registration may have increased the risk of methodological flexibility in study selection, quality assessment, and narrative interpretation.

### 4.4. The Missing Link: Functional Validation and Computational Fluid Dynamics (CFD)

Although none of the included studies directly evaluated computational fluid dynamics (CFD) or patient-specific functional modeling, these tools are relevant to the translational interpretation of the present findings because they may help bridge the gap between structural reshaping and functional validation. One major gap in the literature is the absence of functional validation beyond geometric reshaping. Both LCR and EMR studies predominantly report bend angles, shape retention, histologic findings, or viability changes, yet none evaluates whether these structural modifications translate into clinically meaningful improvement in nasal airflow. This is a critical limitation, because the relationship between septal geometry and nasal obstruction is not linear and may vary substantially across patients depending on the location of the deviation, the contribution of adjacent structures, and the baseline nasal airflow pattern.

In this context, CFD is particularly relevant because it can move the field from generic reshaping toward function-oriented, individualized planning. Small geometric corrections may be sufficient to improve airflow in some patients, while similar reshaping may have negligible functional effect in others. Therefore, CFD could help identify which cartilage-dominant deformities are likely to benefit from energy-based reshaping, estimate the magnitude and location of correction required, and support patient-specific optimization of treatment strategies [25,26]. By linking geometry to patient-specific airflow consequences, CFD could transform reshaping from a generic biomechanical intervention into a precision-guided functional strategy.

Integrating CFD into future LCR and EMR studies would therefore provide more than a technical adjunct. It would help determine whether modest cartilage reshaping produces meaningful airflow improvement, enable patient-specific prediction of functional benefit, guide parameter selection, and validate reshaping approaches against functional endpoints rather than purely structural ones. Such integration could represent the key step needed to translate these technologies from experimental cartilage manipulation to personalized functional septal surgery. Future studies should combine anatomical imaging, endoscopy, symptom scores, and CFD-derived airflow metrics to define patient-specific indications for septal cartilage reshaping. At the same time, the incorporation of CFD into routine clinical workflows remains limited by practical constraints. Patient-specific CFD requires high-quality imaging, segmentation, technical expertise, computational resources, and additional processing time, all of which may reduce feasibility in rapid, office-based settings. This is particularly relevant because one of the theoretical attractions of EMR and, to a lesser extent, LCR is their potential use as minimally invasive and streamlined interventions. Accordingly, CFD is best viewed at present as a translational and research-enabling tool for treatment planning, validation, and phenotype stratification, rather than as an immediately scalable component of routine clinical practice [27]. Future progress will depend not only on demonstrating its functional value, but also on simplifying and accelerating workflow integration.

### 4.5. Personalized Medicine Perspective

The following considerations should be interpreted as a conceptual and future-oriented translational framework, rather than as evidence directly demonstrated by the included studies. To better conceptualize the potential translational pathway of these technologies, future research may be framed within a “Precision Septoplasty Model”, integrating patient phenotype and symptom burden, anatomical imaging, CFD-based functional assessment, phenotype classification, targeted reshaping strategies, outcome prediction, and patient-specific follow-up. Within such a model, energy-based reshaping would not be used indiscriminately, but rather as a tailored intervention for carefully selected septal phenotypes. From a personalized medicine perspective, the main potential advantage of LCR and EMR is not their use as universal alternatives to conventional septoplasty, but their possible role in tailoring correction to specific patient phenotypes. Nasal septal deviation is a heterogeneous condition in which symptom severity, airflow impairment, cartilage thickness, deformity pattern, and the relative contribution of cartilaginous versus bony components vary substantially between individuals. In this context, energy-based reshaping may be most relevant in carefully selected patients with mild to moderate anterior, cartilage-dominant deviations, in whom limited structural modification could translate into meaningful functional benefit. Future clinical development should therefore move beyond proof-of-concept reshaping and adopt a patient-specific framework integrating anatomical assessment, symptom burden, endoscopy, airflow testing, and computational modeling [28]. Such an approach could help identify which patients are suitable candidates, define the amount and location of reshaping required, and support individualized procedural planning. In this sense, the real translational value of LCR and EMR may lie less in replacing septoplasty as a whole than in enabling more precise, phenotype-oriented correction strategies within personalized functional nasal surgery.

### 4.6. Future Directions Toward Precision Functional Septoplasty

Based on the limitations identified in the current literature, several future research directions can be proposed, although these remain hypothesis-generating rather than evidence-based recommendations. Future progress in this field should move beyond demonstration of cartilage shape change alone and toward an integrated precision framework. Within such a framework, LCR and EMR are more plausibly envisioned as adjunctive tools within multimodal septal reconstruction than as full replacements for conventional septoplasty. First, future studies should define clinically meaningful septal phenotypes, distinguishing cartilage-dominant deviations that may be suitable for reshaping from mixed or osseocartilaginous deformities in which reshaping, if used at all, would more likely serve as an adjunct to conventional septoplasty. Second, anatomical imaging and CFD should be combined to identify patient-specific treatment targets and predict whether limited reshaping is likely to produce measurable airflow benefit. Third, procedural development should focus on personalized dosimetry, device geometry, and ergonomically suitable intranasal delivery systems. Finally, prospective translational studies should incorporate symptom scores, objective airflow assessment, and longitudinal follow-up to determine whether targeted reshaping can become a reproducible component of precision functional nasal surgery [29].

In addition to technical and biological challenges, future clinical translation will also depend on overcoming regulatory and commercialization barriers. Both LCR and EMR currently rely on experimental or semi-customized systems that lack the device standardization, usability validation, and procedural reproducibility required for broad regulatory approval. The path toward clinical adoption will likely require not only demonstration of efficacy and safety, but also the development of commercially viable, standardized intranasal platforms with reproducible dosimetry, ergonomic usability, and quality-controlled manufacturing. These requirements may partly explain why, despite decades of experimental work, neither technology has yet achieved widespread clinical implementation.

## 5. Conclusions

Research on LCR and EMR has spanned more than three decades [30], yet remains predominantly confined to animal models and ex vivo experiments. Both modalities reliably induce cartilage reshaping, but their role in bony or complex osseocartilaginous deviations is likely to be limited to selected adjunctive applications rather than stand-alone correction. Human evidence is limited to a small number of uncontrolled LCR studies, while EMR has not yet been evaluated clinically for septoplasty [12,13,14].

Significant challenges remain, including narrow safety margins, lack of standardized dosimetry, limited understanding of long-term biological responses, and the absence of clinically suitable intranasal devices. While both techniques hold promise for minimally invasive correction of selected anterior, cartilage-dominant deviations, they are unlikely to replace conventional septoplasty and may be more realistically integrated as adjunctive tools within hybrid treatment strategies. Their most realistic future role may lie in hybrid treatment strategies, in which conventional septoplasty addresses the bony or structural component and energy-based reshaping is used selectively for cartilage-dominant refinement.

Their future clinical relevance will likely depend on a shift from purely structural reshaping toward function-driven, patient-specific strategies. In particular, progress will require improved instrumentation, rigorous translational studies, long-term human data, and functional validation through individualized assessment tools such as computational fluid dynamics. In this context, computational fluid dynamics may provide the missing link between anatomical reshaping and patient-specific respiratory benefit. Future studies should therefore move from generic cartilage deformation toward phenotype-driven, function-oriented septal correction. Within such a precision framework, LCR and EMR may ultimately find a role not as universal substitutes for septoplasty, but as adjunctive components of personalized and potentially hybrid functional nasal septal surgery. The translational challenge is therefore no longer simply to reshape cartilage, but to demonstrate that controlled reshaping can produce predictable, patient-specific functional benefit. Achieving this goal will require not only better technology and more standardized dosimetry, but also clinically feasible trial designs and workflow models that can bridge the gap between experimental precision and real-world otolaryngologic practice.

### Clinical Implications

Energy-based reshaping may eventually expand the therapeutic options available to rhinologists by offering office-based, minimally invasive correction of mild cartilaginous deviations. However, their current applicability remains limited by the scarcity of human data, the inability to independently correct osseocartilaginous deformities, the incomplete understanding of long-term effects, the lack of dedicated intranasal devices, the cost constraints associated with LCR, and the lack of commercial availability of EMR systems. For now, LCR and EMR should be considered adjunctive technologies rather than alternatives to conventional septoplasty, potentially useful for selected contour corrections, postoperative refinements, or hybrid strategies targeting the cartilaginous component of deviation when conventional structural correction remains necessary. This potential role is likely to be adjunctive and hybrid rather than substitutive, particularly in patients with mixed deformity patterns requiring multimodal correction.

## Figures and Tables

**Table 1:** Summary of critical appraisal of included studies.

Author, Year	Design/Model	Appraisal Tool	Overall Appraisal	Main Concerns
Slater et al., 2005 [6]	Porcine (ex vivo)	Domain-based ex vivo framework	Moderate-to-high concerns	No clear tissue control group, sample/replicate reporting limited, no blinding reported
Youn et al., 2005 [7]	Porcine (ex vivo)	Domain-based ex vivo framework	Moderate concerns	Sample-size reporting limited; no blinding reported
Karamzadeh et al., 2001 [8]	Porcine (ex vivo)	Domain-based ex vivo framework	Moderate concerns	Limited replicate reporting; bench design with surrogate outcomes only
Karam et al., 2006 [9]	Rabbit (in vivo)	SYRCLE RoB tool	Unclear-to-high risk of bias	Randomization, concealment, housing and blinding insufficiently reported
Wong et al., 2000 [10]	Porcine (ex vivo)	Domain-based ex vivo framework	Moderate concerns	No blinding reported; limited direct clinical applicability
Li et al., 2007 [11]	Rabbit (ex vivo)	Domain-based ex vivo framework	Moderate concerns	Control and replicate reporting not fully detailed; surrogate laboratory endpoints
Bourolias et al., 2008 [12]	Human case series	JBI Case Series	Moderate concerns	Uncontrolled design, unclear consecutive recruitment, short follow-up
Sobol et al., 2010 [13]	Human case series/conference report	JBI Case Series	High concerns	Sparse reporting, unclear denominator consistency, vague outcome definitions, limited statistical detail
Leclère et al., 2010 [14]	Human case series	JBI Case Series	Moderate concerns	Small sample, uncontrolled design, short follow-up
Protsenko et al., 2006 [15]	Porcine (ex vivo)	Domain-based ex vivo framework	Moderate concerns	No blinding reported; mechanistic outcomes only
Wu et al., 2009 [16]	Rabbit (ex vivo)	Domain-based ex vivo framework	Moderate-to-high concerns	Conference-format reporting, limited replicate detail, no blinding reported
Karimi et al., 2010 [17]	Rabbit (ex vivo)	Domain-based ex vivo framework	Moderate concerns	Replicate reporting limited; indirect relevance to clinical reshaping
Lim et al., 2011 [18]	Rabbit (ex vivo)	Domain-based ex vivo framework	Moderate concerns	No blinding reported; ex vivo mechanics only
Protsenko et al., 2011 [19]	Rabbit (ex vivo)	Domain-based ex vivo framework	Moderate concerns	No blinding reported; long-term viability assessed only in culture model
Wu et al., 2011 [20]	Rabbit (ex vivo)	Domain-based ex vivo framework	Moderate-to-high concerns	Pilot-style reporting, limited replicate/statistical detail, surrogate mechanistic endpoints
Wu et al., 2011 [21]	Rabbit (ex vivo)	Domain-based ex vivo framework	Moderate concerns	No blinding reported; ex vivo outcome only
Kuan et al., 2014 [22]	Rabbit (ex vivo)	Domain-based ex vivo framework	Moderate concerns	Mechanistic ex vivo design; no blinding reported
Jahankir et al., 2025 [23]	Goat (ex vivo)	Domain-based ex vivo framework	Moderate concerns	Ex vivo design, no blinding reported, short-term retention only

**Table 2:** Laser-mediated cartilage reshaping (LCR).

Author, Year	Model	Sample Size (*n*)	Energy Parameters	Key Findings	Follow-Up/Assessment Timeframe
Slater et al., 2005 [6]	Porcine (ex vivo)	NR	Nd:YAG laser λ = 1.32 μm, 4 W, for 13″.	Acoustic monitoring feasible during reshaping	Immediate/intra-procedural assessment
Youn et al., 2005 [7]	Porcine (ex vivo)	NR	Nd:YAG λ = 1.32 μm, 6 W, for 6″.	Polarization-sensitive optical coherence tomography (PS-OCT) detects dehydration during reshaping	Immediate/intra-procedural assessment
Karamzadeh et al., 2001 [8]	Porcine (ex vivo)	4	Nd:YAG laser λ = 1.32 μm, 10 W, for 10″.	Laser-treated regions: cell viability maintained, but matrix loss in histologic specimen.	Immediate post-treatment assessment
Karam et al., 2006 [9]	Rabbit (in vivo)	18	Nd:YAG laser λ = 1.32 μm, 4–8 W, for 6–16″.	Dose-dependent reshaping; late structural failure	Long-term in vivo assessment
Wong et al., 2000 [10]	Porcine (ex vivo)	3	Nd:YAG laser λ = 1.32 μm, 25 W, for 5–8.3–12.2″.	Proteoglycan synthesis decreased with successive laser exposure	Post-treatment biochemical assessment
Li et al., 2007 [11]	Rabbit (ex vivo)	NR	Nd:YAG laser λ = 1.32 μm, 4–6–8 W, 4–6–8–10–12–16″.	No safe dosimetry without tissue damage. Direct relationship between laser dosimetry and tissue damage.	Acute post-treatment damage assessment
Bourolias et al., 2008 [12]	Human case series	67	Er:Glass λ = 1.56 μm, 4.1 W. Mean operative time: 35′.	Improved NOSE and airflow (short-term)	Postoperative short-term clinical follow-up
Sobol et al., 2010 [13]	Human case series/conference report	170	Er:Glass λ = 1.56 μm, up to 3 W.	Positive results in 95% of patients (2 years follow-up)	2-year follow-up
Leclère et al., 2010 [14]	Human case series	12	Er:Glass λ = 1.56 μm, 500 W, 3.3 ms. Mean operative time: 20′.	Improved NOSE, flow and resistance measurements.	Postoperative clinical follow-up (duration not clearly extractable from accessible abstract)

NR = not reported.

**Table 3:** Electromechanical Reshaping (EMR).

Author, Year	Model	Sample Size (*n*)	Energy Parameters	Key Findings	Follow-Up/Assessment Timeframe
Protsenko et al., 2006 [15]	Porcine (ex vivo)	NR	~1 mV, 60″: increasing 1 V/s and then reducing 1 V/s. 1–10 V, 30″–6′.	Electric field in a mechanically deformed cartilage specimen accelerates the relaxation of internal stress with sustained shape change.	Immediate post-EMR mechanical assessment
Wu et al., 2009 [16]	Rabbit (ex vivo)	NR	Parameters: 1, 2, 4, 6, 8 V, for 2′.	Voltage-dependent reshaping. Number and size of needle electrodes increase risk of trauma.	Immediate post-EMR shape assessment
Karimi et al., 2010 [17]	Rabbit (ex vivo)	NR	1st group: 2 V, for 2′. 2nd group: 6 V, for 2′.	Voltage-dependent reshaping.	Immediate post-EMR bend-angle assessment
Lim et al., 2011 [18]	Rabbit (ex vivo)	52	Parameters: 2, 4, 5, 6, 8 V, for 2′.	Reduced stiffness above 2 V.	Immediate post-EMR mechanical assessment
Protsenko et al., 2011 [19]	Rabbit (ex vivo)	NR	3, 4, 5, and 6 V and 1, 2, and 3′, respectively.	Viability loss is proportional to reshaping.	Culture follow-up up to 64 days
Wu et al., 2011 [20]	Rabbit (ex vivo)	NR	Reshaping group: 4 V for 4′, 6 V for 1′, 6 V for 2′. Tissue damage group: 8 V for 4′, 10 V for 5′.	pH change predicts tissue damage	Immediate post-EMR pH mapping
Wu et al., 2011 [21]	Rabbit (ex vivo)	200	Parameters: 1, 2, 4, 6, 8 V, applied for 1′–2′–4′	Clinically relevant reshaping ≥ 4 V	Immediate post-EMR shape assessment
Kuan et al., 2014 [22]	Rabbit (ex vivo)	5–6 per parameter set (and 3 per parameter set for hue analysis)	Shape group: 4 V, 4′; 6 V, 1′–2′–4′; 8 V, 1′. Tissue damage group: 4 V, 4′; 6 V, 1′–2′–4′; 8 V, 1′.	Charge-dependent pH gradients	Immediate post-EMR pH/transition-zone assessment
Jahankir et al., 2025 [23]	Goat (ex vivo)	NR	Flat titanium electrodes: 20 mA, 35 mA, 45 mA, and 50 mA for 15′. Titanium strip electrodes: 10 mA, 15 mA, 20 mA, and 30 mA for 15′.	Strip electrodes improve shape retention	Short-term ex vivo retention/structural assessment

NR = not reported.

## Data Availability

No new data were created or analyzed in this study. Data sharing is not applicable to this article.

## Supplementary Materials

The following supporting information can be downloaded at: https://www.mdpi.com/article/10.3390/jpm16060304/s1. Table S1: Detailed critical appraisal matrix of included studies; File S1: Database-specific search strategies; File S2: PRISMA 2020 checklist. Reference [31] is cited in Supplementary Materials.

## Abbreviations

The following abbreviations are used in this manuscript:

CFD	Computational fluid dynamics
CI	Consecutive inclusion
Clin	Clinical information reported
CM	Condition measured in a standard, reliable way
CompI	Complete inclusion
Demo	Demographics reported
EMR	Electromechanical reshaping
ER	Erbium-doped glass fiber laser
IC	Inclusion criteria clearly stated
JBI	Joanna Briggs Institute
LCR	Laser-mediated cartilage reshaping
NA	Not available
Nd:YAG	Neodymium-doped yttrium aluminum garnet
NOSE	Nasal Obstruction Symptom Evaluation
NSD	Nasal septal deviation
Outcomes	Outcomes and follow-up clearly reported
PICOS	Population, Intervention, Comparator, Outcomes, Study type
PRISMA	Preferred Reporting Items for Systematic Reviews and Meta-Analyses
PS-OCT	Polarization-sensitive optical coherence tomography
RoB	Risk of bias
SeqGen	Sequence generation
Site	Site or clinic demographics described
Stats	Appropriate statistical analysis
SYRCLE	Systematic Review Centre for Laboratory Animal Experimentation
U	Unclear
VM	Valid methods used for condition identification
Y	Yes
L	Low risk
H	High risk
