# Supplementary material for: From Conventional Septoplasty to Patient-Specific Cartilage Reshaping: A Systematic Review of Laser-Mediated and Electromechanical Approaches
Source: J Pers Med. 2026 Jun 3;16(6):304. doi: 10.3390/jpm16060304 (PMC13301324; doi:10.3390/jpm16060304)
Supplement: Supplementary file 1 [file jpm-16-00304-s001.zip › jpm-4343610 - Supplementary File S1. Database-specific search strategies.pdf]

Supplementary File S1. Database-specific search strategies

The electronic search strategy was developed from the following core concept: (cartilage AND (reshaping OR remodeling OR molding OR contouring)) AND (laser OR electromechanical OR thermal OR radiofrequency OR "energy-based"). The syntax was adapted to each database as follows:

PubMed: (cartilage[Title/Abstract] AND (reshaping[Title/Abstract] OR remodeling[Title/Abstract] OR molding[Title/Abstract] OR contouring[Title/Abstract])) AND (laser[Title/Abstract] OR electromechanical[Title/Abstract] OR thermal[Title/Abstract] OR radiofrequency[Title/Abstract] OR "energy-based"[Title/Abstract])

Scopus: TITLE-ABS-KEY (cartilage AND (reshaping OR remodeling OR molding OR contouring) AND (laser OR electromechanical OR thermal OR radiofrequency OR "energy-based"))

Web of Science: TS=(cartilage AND (reshaping OR remodeling OR molding OR contouring) AND (laser OR electromechanical OR thermal OR radiofrequency OR "energy-based"))
